# Crystal structure, Hirshfeld surface analysis and energy frameworks of 1-[(*E*)-2-(2-fluoro­phen­yl)diazan-1-yl­idene]naphthalen-2(1*H*)-one

**DOI:** 10.1107/S2056989024000227

**Published:** 2024-01-12

**Authors:** Hibet Errahmane Meroua Akkache, Noudjoud Hamdouni, Ali Boudjada, Mohamed larbi Medjroubi, Assia Mili, Olivier Jeannin

**Affiliations:** a Laboratoire de Cristallographie, Département de Physique, Université Mentouri-Constantine, 25000 Constantine, Algeria; b Unité de Recherche de Chimie de l’Environnement et Moléculaire Structurale, Faculté du Sciences Exactes, Université de Constantine 1, 25000 Constantine, Algeria; c UMR 6226 CNRS–Université Rennes 1, ‘Sciences Chimiques de Rennes’, Equipe ‘Matière Condensée et Systèmes Electroactifs’, Bâtiment 10C Campus de Beaulieu, 263 Avenue du Général Leclerc, F-35042 Rennes, France; Universidade Federal do ABC, Brazil

**Keywords:** azo compounds, 2-naphthols, crystal structure, Hirshfeld surface calculations, two-dimensional fingerprint plot, energy frameworks

## Abstract

The title compound, C_16_H_11_N_2_OF, is a member of the azo dye family. The dihedral angle subtended by the benzene ring and the naphthalene ring system measures 18.75 (7)°, indicating that the compound is not perfectly planar. An intra­molecular N—H⋯O hydrogen bond occurs between the imino and carbonyl groups. In the crystal, the mol­ecules are linked into inversion dimers by C—H⋯O inter­actions. Aromatic π–π stacking between the naphthalene ring systems lead to the formation of chains along [001]. A Hirshfeld surface analysis was undertaken to investigate and qu­antify the inter­molecular inter­actions. In addition, energy frameworks were used to examine the cooperative effect of these inter­molecular inter­actions across the crystal, showing dispersion energy to be the most influential factor in the crystal organization of the compound.

## Abstract 2

In the crystal, mol­ecules of the title compound are linked into infinite sinusoidal chains along the [001] direction. The study demonstrated that dispersion energy was the most influential factor in the crystal organization of the compound.

## 1. Chemical context

In dye chemistry, azo dyes are produced in the most significant qu­anti­ties (Benkhaya *et al.*, 2020 ▸). Azo compounds are commonly used in various industrial applications, including as colourants (Mohammadi *et al.*, 2015 ▸) and pigments (Ramugade *et al.*, 2019 ▸; Vafaei *et al.*, 2012 ▸) and in printing (Nawwar *et al.*, 2020 ▸). Azo dyes are generally used in the leather, food, and cosmetics industries because they have bright colours and good stability. Apart from this, they have been widely employed in a variety of areas including the food (Yamjala *et al.*, 2016 ▸) and cosmetics industries (Leulescu *et al.*, 2021 ▸) and as metal–organic frameworks (MOFs) (Ayati *et al.*, 2016 ▸), covalent–organic frameworks (COFs) (Xue *et al.*, 2023 ▸), corrosion inhibitors for iron (Madkour *et al.*, 2018 ▸), catalysis (Liu *et al.*, 2016 ▸), non-linear optics (Kato *et al.*, 1994 ▸) and fibre optics (Kavitha *et al.*, 2022 ▸). In addition to this, azo dyes have been found to have biological, biomedical, and pharmacological applications, such as in DNA binding and anti­oxidants (Qamar *et al.*, 2019 ▸), drug design (Demirçalı & Topal, 2023 ▸), and virology (Meng *et al.*, 2021 ▸). However, it is important to understand that some azo dyes can harm human health and the environment. This is because of their potential to release carcinogenic aromatic amines when they undergo degradation processes triggered by bacteria or sunlight (Golka *et al.*, 2004 ▸)*.* Following our inter­est in azo dyes, we present the crystal structure of a new azo compound 1-[(*E*)-2-(2-fluoro­phen­yl)di­azan-1-yl­idene]naphthalen-2(1*H*)-one. 
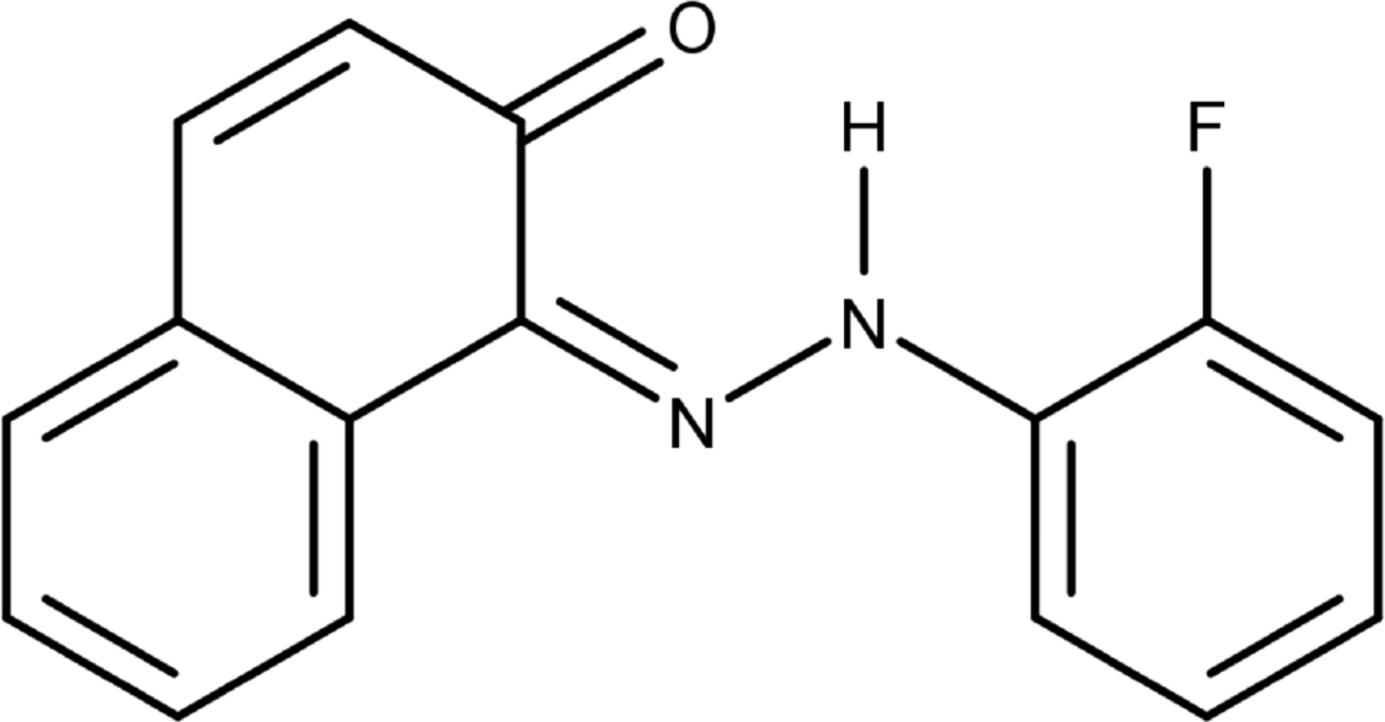


## 2. Structural commentary

**Figure 1:**
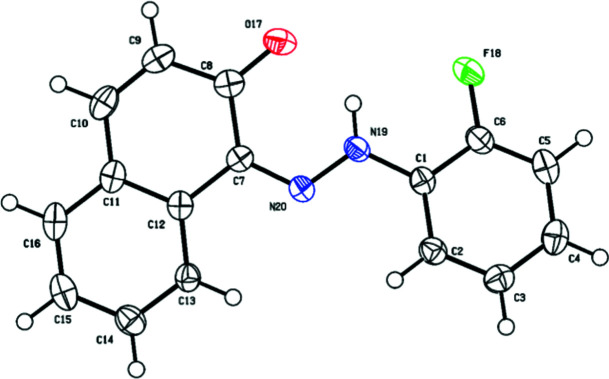
The mol­ecular structure of the title compound with the atom labelling and displacement ellipsoids drawn at the 50% probability level.

The structure of the title compound is illustrated in Fig. 1 ▸. The N19—N20 [1.310 (2) Å] and C8—O17 [1.264 (3) Å] bond lengths indicate that the compound adopts the neutral hydrazo tautomer form upon crystallization. This is common when an OH group is in the *ortho*-position relative to the azo group, leading to a proton being transferred from the naphthol group to the azo group (Benaouida *et al.*, 2023 ▸; Bougueria *et al.*, 2021 ▸). The inter­nal alternate angles at N19 and N20 are identical within experimental error with an average value of 118.25 (2)°. This is not observed in the isotypic product (Bougueria *et al.*, 2017 ▸). Bond lengths are within normal ranges and resemble those observed in isotypic crystal structures (Bougueria *et al.*, 2017 ▸). The naphthol and benzene rings, which are connected to the hydrazo group, are not perfectly planar. The dihedral angle between these rings is 18.75 (7)°. However, in the isotopic variant of the mol­ecule, this angle was slightly smaller at 15.33 (7)° (Bougueria *et al.*, 2017 ▸). An intra­molecular hydrogen bond (Table 1 ▸) contributes to the mol­ecular stability. The most significant exocyclic angle C7—C8—O17 [121.5 (2)°] adjacent to the C8—O17 bond could be attributed to the critical inter­action between the O17 and H21 atoms. The smallest exocyclic angle C1—C6—F18 [117.4 (2)°] adjacent to the C6—F18 bond may be due to an attractive inter­action between fluorine and hydrogen.

## 3. Supra­molecular features

**Figure 2:**
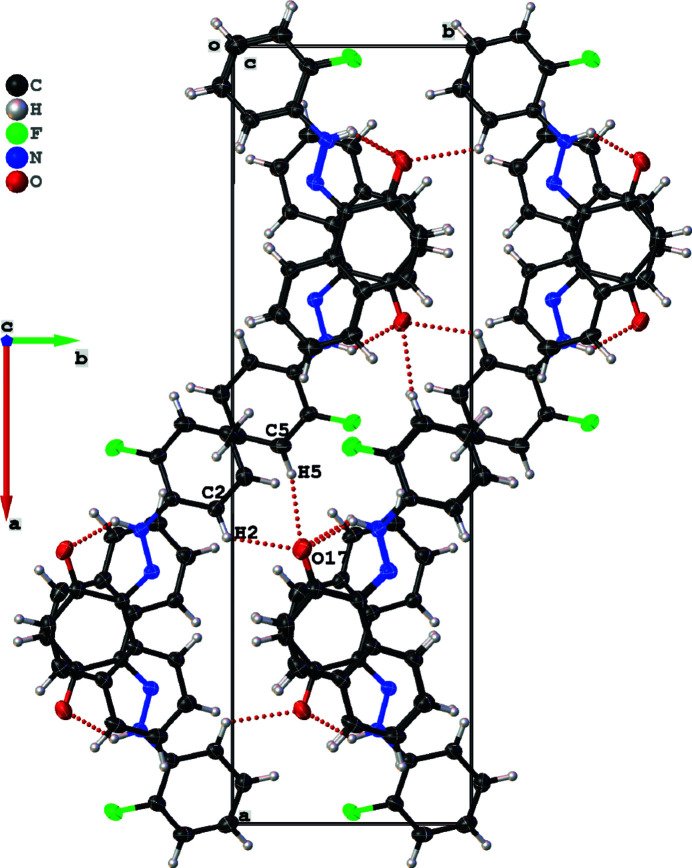
A view along the *c* axis of the crystal packing.

**Figure 3:**
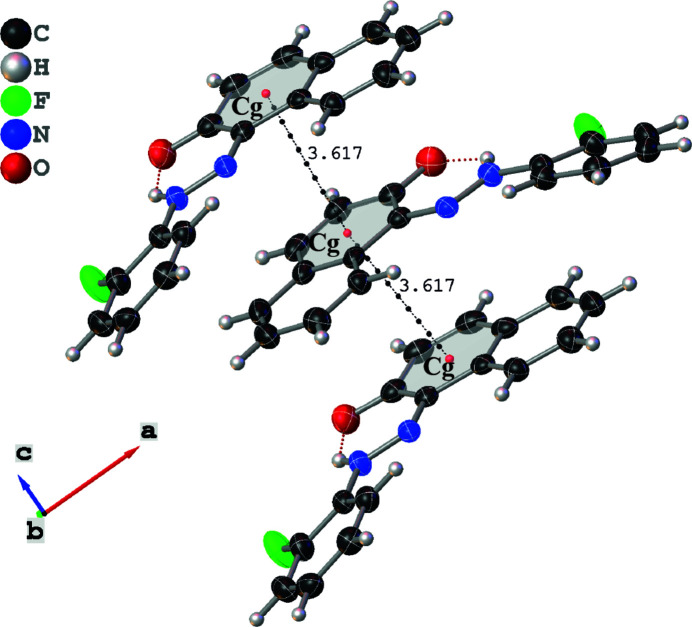
π–π stacking inter­actions in the title compound. Dotted black lines indicate *Cg*⋯*Cg* contacts.

In the crystal, the mol­ecules are linked by inter­molecular C—H⋯O hydrogen bonds (Table 1 ▸), see Fig. 2 ▸. Cohesion of the crystal is enhanced by the presence of parallel displaced π–π stacking inter­actions (Fig. 3 ▸), the most significant of which is between naphthalene ring systems [*Cg*⋯*Cg*( 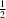 − *x*, *y*,1/2 + *z*) = 3.6171 (4) Å where *Cg* is the centroid of the C7–C12 ring], forming sinusoidal chains along the *c*-axis direction.

## 4. Hirshfeld surface analysis (HS), inter­action energies and energy frameworks

**Figure 4:**
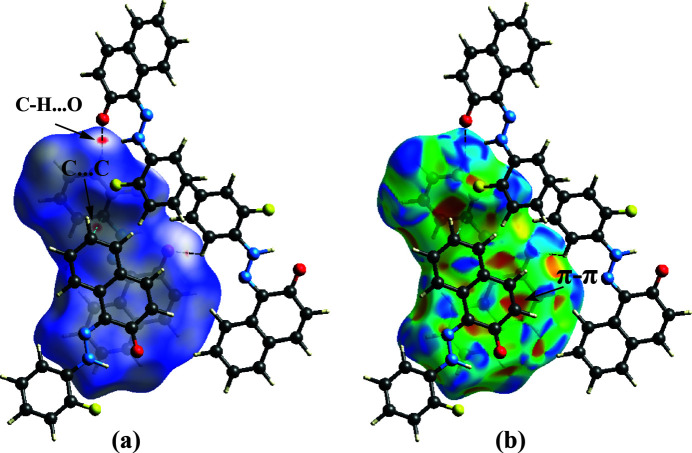
Hirshfeld surface mapped over (*a*) *d*_norm_ and (*b*) shape-index.

**Figure 5:**
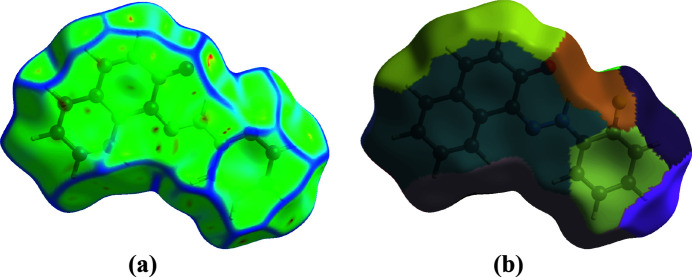
(*a*) Curvedness and (*b*) fragment patch along [001].

**Figure 6:**
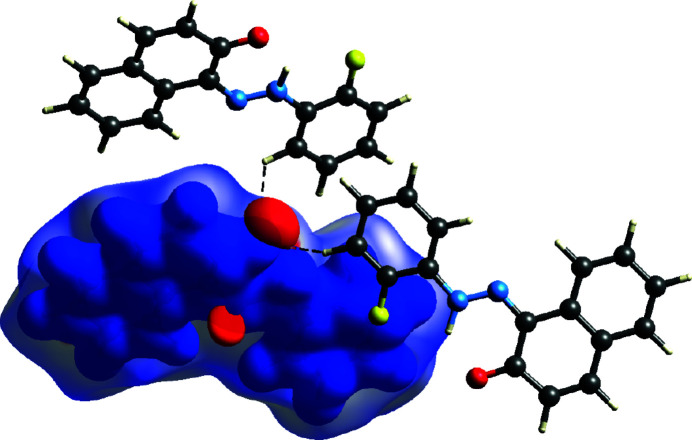
Electrostatic potential mapped on the Hirshfeld surface along [001].

The weak inter­molecular inter­actions within the crystal structure were examined by analysing Hirshfeld surfaces (Spackman & Jayatilaka, 2009 ▸). The associated 2D fingerprint plots (Spackman & McKinnon, 2002 ▸) were drawn using *CrystalExplorer21* (Spackman *et al.*, 2021 ▸). Measuring and inter­preting the inter­molecular inter­actions within the crystal packing is visualized through normalized contact distance (*d*_norm_). In this context, white denotes contacts with distances equal to the van der Waals (vdW) radii. Connections that are short of the vdW radii are represented in red, while those that exceed the vdW radii are shown in blue. In Fig. 4 ▸ *a*, dark-red spots represent strong inter­molecular C—H⋯O hydrogen bonds and light-red spots represent C⋯C close inter­actions. In addition, the shape-index is used to identify complementary hollows (red) and bumps (blue) where two mol­ecular surfaces touch one another (Spackman & Jayatilaka, 2009 ▸). As depicted in Fig. 4 ▸ *b*, the two sides of the mol­ecule inter­act differently with adjacent mol­ecules. This includes π–π stacking, represented by adjacent red and blue triangles (McKinnon *et al.*, 2004 ▸). Curvedness is a tool for pinpointing planar stacking configurations and how neighbouring mol­ecules inter­act (Spackman & Jayatilaka, 2009 ▸). Fig. 5 ▸ *a* shows relatively large green planes in the benzene and naphthalene rings separated by blue edges. These green planes give us an idea of the flatness of complexes, and the fragment patch (Fig. 5 ▸ *b*) is designed to indicate the nearest neighbouring mol­ecule (Spackman *et al.*, 2021 ▸). The electrostatic potential was mapped using *TONTO* (Spackman & Jayatilaka, 2009 ▸), integrated into *CrystalExplorer*, with the STO-3G basis/Hartree–Fock functio. The contacts are discernible as areas of electropositivity (blue) and electronegativity (red) that exhibit a complementary relationship (Spackman *et al.*, 2008 ▸). These short contacts correspond to C—H⋯O. Blue and red areas around the atoms denote hydrogen-bond donors and acceptors, respectively. These colours indicate the positive and negative electrostatic potentials in Fig. 6 ▸.

**Figure 7:**
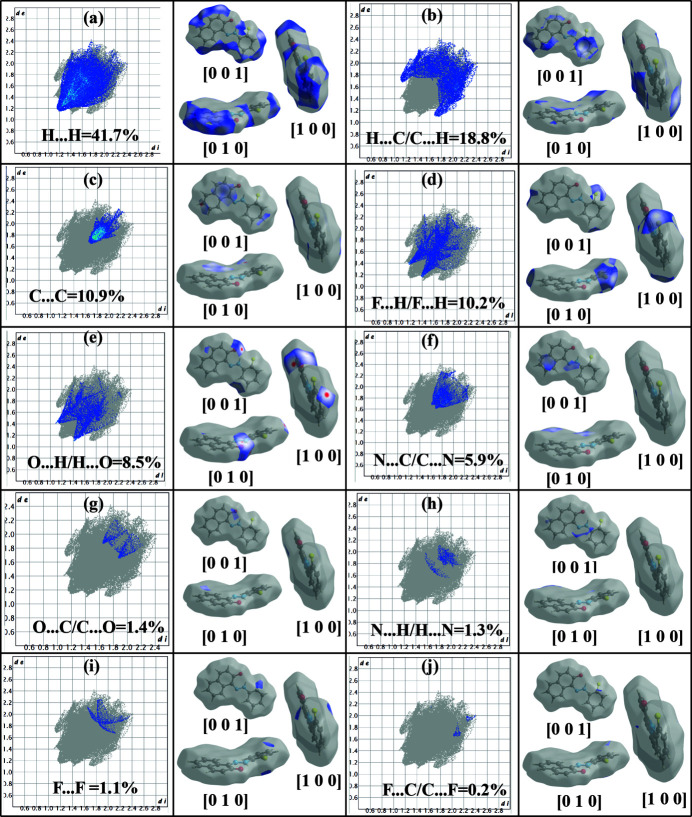
Two-dimensional fingerprint plots for the title compound showing the contributions of different types of inter­actions: (*a*) H⋯H, (*b*) C⋯H/H⋯C, (*c*) C⋯C, (*d*) H⋯F/F⋯H, (*e*) O⋯H/H⋯O, (*f*) C⋯N/N⋯C, (*g*) N⋯H/H⋯N, (*h*) F⋯F, (i) F⋯C/C⋯F, (*j*) F⋯C/C⋯F.

The proportional contribution of the contacts over the surface is visualized in the fingerprint plots with the Hirshfeld surface of the contribution (Table 2 ▸). The fingerprint plots of the H⋯H contacts, which represent the most significant contribution to the Hirshfeld surfaces at 41.7%, show a distinct pattern with a minimum value of *d*_e_ = *d*_i_ ≃1.2 Å (Fig. 7 ▸ *a*). The contribution of the C⋯H/H⋯C contacts appears as the second largest region of the fingerprint plot, heavily concentrated on the edges with *d*_e_ + *d*_i_ ≃2.8 Å and an overall Hirshfeld surface contribution of 18.8% (Fig. 7 ▸ *b*). The C⋯C contacts occupy 10.9% of the Hirshfeld surface with *d*_e_ + *d*_i_ ≃ 1.7 Å. Bonds are observed around light-red spots among these contacts (Fig. 7 ▸ *c*). The H⋯F/F⋯H contacts contribute 10.2% of the Hirshfeld surface with *d*_e_ + *d*_i_ ≃ 2.6 Å (Fig. 7 ▸ *d*). The O⋯H/H⋯O contacts, with a contribution of 8.5% and *d*_e_ + *d*_i_ ≃ 2.5 Å, appear as dark-red spots on the Hirshfeld surfaces mapped over *d*_norm_ (Fig. 7 ▸ *e*). The percentage contribution of the C⋯N/N⋯C contacts is 5.9% with *d*_e_ + *d*_i_ ≃ 3.3 Å (Fig. 7 ▸ *f*) while the O⋯C/C⋯O inter­action, with a contribution of 1.4% is in the form of symmetrical claws with the two ends pointing towards pairs at *d*_e_ + *d*_i_ ≃ 3.5 Å and *d*_e_ + *d*_i_ ≃ 3.6 Å (Fig. 7 ▸ *g*). The N⋯H/H⋯N (Fig. 7 ▸ *h*), F⋯F (Fig. 7 ▸ *i*) and F⋯C/C⋯F (Fig. 7 ▸ *j*) inter­actions are the weakest with contributions of 1.3%, 1.1% and 0.2% and *d*_e_ + *d*_i_ ≃ 3.6 Å, 3.8 Å and 3.5 Å, respectively.

**Figure 8:**
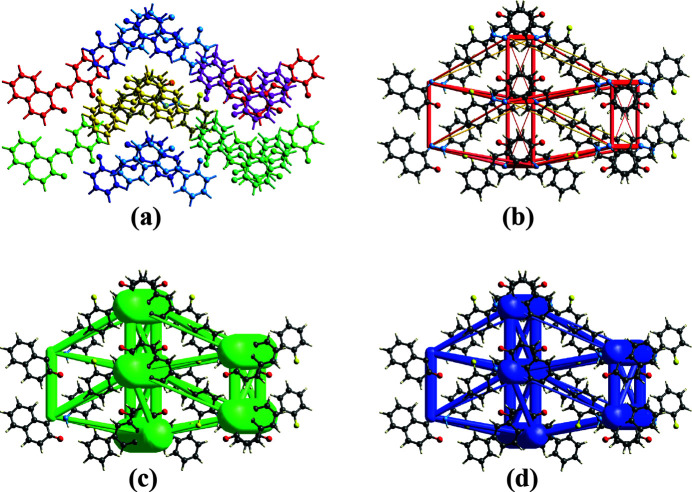
(*a*) Inter­actions between the reference mol­ecule and the mol­ecules present in a 3.8 Å cluster around energy frameworks built for (*b*) *E*_ele_ (red cylinders), (*c*) *E*_dis_ (green cylinders) and *E*_tot_ (blue cylinders) along [001].

The total inter­molecular energy *E*_tot_ (kJ mol^−1^) is the sum of four main energy components: electrostatic, polarisation, dispersion and exchange repulsion (Mackenzie *et al.*, 2017 ▸; Spackman *et al.*, 2021 ▸). The calculation was performed for a cluster of mol­ecules within a 3.8 Å radius surrounding the selected mol­ecule (Fig. 8 ▸ *a*) using the HF/3-21G energy model in conjunction with adjustment coefficients for energy models that have been benchmarked to determine *E*_tot_ (kJ mol^−1^): *K*_ele_=1.019, *K*_dis_= 0.651, *K*_rep_= 0.901. The inter­action energies, as determined by the energy model, suggest that inter­actions in the crystal are significantly influenced by dispersion components (Table 3 ▸). The inter­action between the selected mol­ecule and the symmetry-related mol­ecule at −*x* + 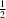 , *y*, *z* + 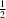 (coloured yellow) is the most important inter­action between neighbouring mol­ecules, with energy: *E*_ele_ = −10.3, *E*_pol_ = −2.7, *E*_dis_ = −75.0, *E*_rep_ = 37.4 and *E*_tot_ = −49.5 kJ mol^−1^. Using energy frameworks (Turner *et al.*, 2015 ▸) built for *E*_ele_ (red cylinders) Fig. 8 ▸ *b*, *E*_dis_ (green cylinders) Fig. 8 ▸ *c*, and *E*_tot_ (blue cylinders) Fig. 8 ▸ *d*, the energies between mol­ecular pairs are represented as cylinders joining the centroids of pairs of mol­ecules. The diameter of these cylinders is adjusted to reflect the degree of change in the inter­action.

## 5. Database survey

A search of the Cambridge Structural Database (CSD; Version 2023.2.0, last update September 2023; Groom *et al.*, 2016 ▸) for 1-phenyl­azo-2-naphthol derivatives revealed that numerous azo-2-naphthol compounds with similar structures have been synthesized using various aromatic primary amines. Examples include (*E*)-1-(3-chloro­phen­yl)-2-(2-oxidonaphthalen-1-yl)diazen-1-ium (AFOJUC; Benosmane *et al.*, 2013 ▸), 1-[(*E*)-2-(5-chloro-2 hy­droxy­phen­yl) hydrazin-1-yl­idene]naphthalen-2(1*H*)-one (UVIDOV; Bougueria *et al.*, 2021 ▸), (*E*)-1-(4-fluoro­phen­yl)-2-(2-oxidonaphthalen-1-yl)diazenium (RAHHIU; Bougueria *et al.*, 2017 ▸), (1*Z*)-naphthalene-1,2-dione 1-[(2-fluoro­phen­yl)hydrazone] (OGUXAP, OGUXAP01, OGUXAP02 and OGUXAP03; Gilli *et al.*, 2002 ▸), (*E*)-1-[2-(3-nitro­phen­yl)hydrazinyl­idene]naphthalen-2(1*H*)-one (FIFCEG; Benaouida *et al.*, 2023 ▸), 1-(phenyl­azo)-2-naphthol (JARPEX; Olivieri *et al.*, 2002 ▸), (*Z*)-1-(2-phenyl­diazen-2-ium-1-yl)naphthalen-2-olate (TIFTEJ01; Benosmane *et al.*, 2015 ▸). All these compounds belong to the azo dyes family and share a common base structure – a benzene ring and a naphthalene ring system linked with an oxygen in the *ortho* position relative to the azo group. This shared structure has almost the same properties. For instance, the azo group contributes to the vivid colors of these dyes, while the specific arrangement of the rings can influence their stability and reactivity.

## 6. Synthesis and crystallization

The title compound was synthesised by two successive reactions, diazo­tization and coupling. 3-Amino­benzaldehyde (0.02 mol) was treated in 6 ml of 12*M* HCl and NaNO_2_ (0.0214 mol) in 8 ml of water for 30 min. To the solution obtained, a solution of naphthalene-2-ol was added dropwise as a coupler where the structural nature of the coupler determined the colour and mol­ecular structure of the C_18_H_16_N_2_O_3_ monomer. The orange–red powder obtained was recrystallized from pentane, leading to prismatic air-stable crystals.

## 7. Refinement details

Crystal data, data collection and structure refinement details are summarized in Table 4 ▸. The H atoms were included in calculated positions and refined as riding: N—H = 0.88 Å, C—H = 0.95 Å with *U*_iso_(H) = 1.2*U*_eq_(N,C).

## Supplementary Material

Crystal structure: contains datablock(s) I. DOI: 10.1107/S2056989024000227/ee2002sup1.cif

Structure factors: contains datablock(s) I. DOI: 10.1107/S2056989024000227/ee2002Isup2.hkl

Supporting information file. DOI: 10.1107/S2056989024000227/ee2002Isup3.cml

CCDC reference: 2323843

Additional supporting information:  crystallographic information; 3D view; checkCIF report

## Figures and Tables

**Table 1:** Hydrogen-bond geometry (Å, °)

*D*—H⋯*A*	*D*—H	H⋯*A*	*D*⋯*A*	*D*—H⋯*A*
N19—H21⋯O17	0.88	1.84	2.541 (3)	135
C2—H22⋯O17^i^	0.95	2.63	3.242 (3)	122
C5—H25⋯O17^ii^	0.95	2.64	3.539 (3)	159

Symmetry codes: (i) 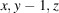 ; (ii) 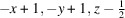 .

**Table 2:** Percentage contributions of various inter­molecular inter­actions to the Hirshfeld surface of the title compound

Contact	Contribution	Contact	Contribution
F⋯F	1.1	N⋯C/C⋯N	5.9
F⋯H/H⋯F	10.2	N⋯H/H⋯N	1.3
F⋯C/C⋯F	0.2	H⋯H	41.7
O⋯H/H⋯O	8.5	H⋯C/C⋯H	18.8
O⋯C/C⋯O	1.4	C⋯C	10.9

**Table 3:** Inter­action energies (kJ mol^−1^) between a reference mol­ecule and its neighbours. *N* is the number of equivalent neighbours, and *R* is the distance between mol­ecular centroids (mean atomic position) in Å. The colours identify mol­ecules in Fig. 8 ▸ *a*, with the reference mol­ecule shown in grey.

Colour	*N*	Symmetry	*R*	Electron density	*E*_ele_	*E*_pol_	*E*_dis_	*E*_rep_	*E*_tot_
Red	2	*x* + 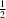 , −*y*, *z*	11.92	HF/3–21G	−5.2	−1.1	−10.7	4.7	−11.8
Yellow	2	−*x* + 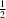 , *y*, *z* + 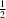	5.00	HF/3–21G	−10.3	−2.7	−75.0	37.4	−49.5
Green	2	*x* + 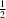 , −*y*, *z*	13.06	HF/3–21G	2.3	−0.5	−7.2	0.0	−4.5
Lime	2	−*x*, −*y*, *z* + 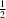	10..67	HF/3–21G	−3.4	−1.0	−17.8	10.8	−11.5
Aqua	2	*x*, *y*, *z*	7.24	HF/3–21G	−7.4	−3.7	−24.0	11.4	−22.3
Indigo	2	−*x* + 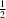 , *y*, *z* + 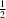	8.80	HF/3–21G	−1.2	−1.8	−15.2	5.3	−11.8
Magenta	2	−*x*, −*y*, *z* + 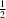	9.23	HF/3–21G	−6.2	−2.1	−9.9	2.9	−14.3

**Table 4:** Experimental details

Crystal data	
Chemical formula	C_16_H_11_FN_2_O
*M*_r_	266.27
Crystal system, space group	Orthorhombic, *P* *c* *a*2_1_
Temperature (K)	150
*a*, *b*, *c* (Å)	23.612 (3), 7.2392 (8), 7.2122 (7)
*V* (Å^3^)	1232.8 (2)
*Z*	4
Radiation type	Mo *K*α
μ (mm^−1^)	0.10
Crystal size (mm)	0.18 × 0.16 × 0.06

Data collection	
Diffractometer	Bruker D8 VENTURE
No. of measured, independent and observed [*I* > 2σ(*I*)] reflections	5289, 2803, 2700
*R*_int_	0.021
(sin θ/λ)_max_ (Å^−1^)	0.650

Refinement	
*R*[*F* ^2^ > 2σ(*F* ^2^)], *wR*(*F* ^2^), *S*	0.044, 0.109, 1.08
No. of reflections	2803
No. of parameters	181
No. of restraints	1
H-atom treatment	H-atom parameters constrained
Δρ_max_, Δρ_min_ (e Å^−3^)	0.32, −0.23
Absolute structure	Flack *x* determined using 1169 quotients [(*I* ^+^)−(*I* ^−^)]/[(*I* ^+^)+(*I* ^−^)] (Parsons *et al.*, 2013 ▸)
Absolute structure parameter	0.0 (3)

Computer programs: *APEX3* and *SAINT* (Bruker, 2015 ▸), *SHELXT2018/2* (Sheldrick, 2015*a* ▸), *SHELXL2018/3* (Sheldrick, 2015*b* ▸), *ORTEP-3 for Windows* and *WinGX* publication routines (Farrugia, 2012 ▸), *Mercury* (Macrae *et al.*, 2020 ▸) and *OLEX2* (Dolomanov *et al.*, 2009 ▸).

## supplementary crystallographic information

### Crystal data

**Table:**

C_16_H_11_FN_2_O	*F*(000) = 552
*M**_r_* = 266.27	*D*_x_ = 1.435 Mg m^−^^3^
Orthorhombic, *P**c**a*2_1_	Mo *K*α radiation, λ = 0.71073 Å
Hall symbol: P 2c -2ac	Cell parameters from 9942 reflections
*a* = 23.612 (3) Å	θ = 2.9–27.5°
*b* = 7.2392 (8) Å	µ = 0.10 mm^−^^1^
*c* = 7.2122 (7) Å	*T* = 150 K
*V* = 1232.8 (2) Å^3^	Prism, orange-red
*Z* = 4	0.18 × 0.16 × 0.06 mm

### Data collection

**Table:**

Bruker D8 VENTURE diffractometer	2700 reflections with *I* > 2σ(*I*)
Radiation source: Enraf–Nonius FR590	*R*_int_ = 0.021
Multilayer monochromator	θ_max_ = 27.5°, θ_min_ = 2.9°
Detector resolution: 7.39 pixels mm^-1^	*h* = 0→30
CCD rotation images, thick slices scans	*k* = −9→9
5289 measured reflections	*l* = −9→9
2803 independent reflections	

### Refinement

**Table:**

Refinement on *F*^2^	Secondary atom site location: dual
Least-squares matrix: full	Hydrogen site location: inferred from neighbouring sites
*R*[*F*^2^ > 2σ(*F*^2^)] = 0.044	H-atom parameters constrained
*wR*(*F*^2^) = 0.109	*w* = 1/[σ^2^(*F*_o_^2^) + (0.0633*P*)^2^ + 0.187*P*] where *P* = (*F*_o_^2^ + 2*F*_c_^2^)/3
*S* = 1.08	(Δ/σ)_max_ < 0.001
2803 reflections	Δρ_max_ = 0.32 e Å^−^^3^
181 parameters	Δρ_min_ = −0.23 e Å^−^^3^
1 restraint	Absolute structure: Flack *x* determined using 1169 quotients [(*I*^+^)-(*I*^-^)]/[(*I*^+^)+(*I*^-^)] (Parsons *et al.*, 2013)
0 constraints	Absolute structure parameter: 0.0 (3)
Primary atom site location: dual	

### Special details

**Table:**

Geometry. All esds (except the esd in the dihedral angle between two l.s. planes) are estimated using the full covariance matrix. The cell esds are taken into account individually in the estimation of esds in distances, angles and torsion angles; correlations between esds in cell parameters are only used when they are defined by crystal symmetry. An approximate (isotropic) treatment of cell esds is used for estimating esds involving l.s. planes.

### Fractional atomic coordinates and isotropic or equivalent isotropic displacement parameters (Å^2^)

**Table:**

	*x*	*y*	*z*	*U*_iso_*/*U*_eq_	
F18	0.48474 (6)	0.5004 (2)	0.4401 (3)	0.0471 (5)	
O17	0.35348 (8)	0.7103 (2)	0.6361 (3)	0.0358 (4)	
N20	0.32550 (7)	0.3449 (2)	0.5164 (3)	0.0229 (4)	
N19	0.37924 (7)	0.3898 (3)	0.5197 (3)	0.0254 (4)	
H21	0.390131	0.49963	0.557997	0.03*	
C1	0.41885 (9)	0.2586 (3)	0.4609 (3)	0.0250 (5)	
C2	0.40634 (9)	0.0731 (3)	0.4374 (3)	0.0283 (5)	
H22	0.369604	0.028034	0.466435	0.034*	
C7	0.28796 (9)	0.4712 (3)	0.5655 (3)	0.0226 (4)	
C4	0.50093 (12)	0.0177 (4)	0.3267 (4)	0.0345 (5)	
H24	0.528484	−0.06543	0.279197	0.041*	
C9	0.25667 (12)	0.7806 (3)	0.6729 (3)	0.0314 (5)	
H27	0.265059	0.901517	0.71613	0.038*	
C14	0.15584 (10)	0.1955 (3)	0.4718 (4)	0.0334 (5)	
H26	0.145461	0.075616	0.430408	0.04*	
C13	0.21242 (9)	0.2415 (3)	0.4897 (3)	0.0269 (5)	
H28	0.240645	0.152688	0.460149	0.032*	
C10	0.20214 (11)	0.7276 (3)	0.6565 (4)	0.0326 (5)	
H29	0.173167	0.813258	0.687556	0.039*	
C3	0.44734 (11)	−0.0462 (3)	0.3718 (4)	0.0328 (5)	
H23	0.438708	−0.173543	0.357452	0.039*	
C8	0.30254 (10)	0.6573 (3)	0.6261 (3)	0.0273 (5)	
C11	0.18627 (10)	0.5474 (3)	0.5941 (3)	0.0273 (5)	
C6	0.47345 (10)	0.3187 (3)	0.4186 (4)	0.0304 (5)	
C12	0.22854 (9)	0.4166 (3)	0.5506 (3)	0.0238 (5)	
C15	0.11400 (10)	0.3244 (4)	0.5144 (4)	0.0364 (6)	
H30	0.07518	0.292542	0.501361	0.044*	
C16	0.12878 (10)	0.4969 (4)	0.5748 (4)	0.0342 (6)	
H31	0.100014	0.5839	0.604186	0.041*	
C5	0.51440 (10)	0.2024 (4)	0.3506 (4)	0.0354 (6)	
H25	0.551065	0.247445	0.320609	0.042*	

### Atomic displacement parameters (Å^2^)

**Table:**

	*U*^11^	*U*^22^	*U*^33^	*U*^12^	*U*^13^	*U*^23^
F18	0.0293 (7)	0.0333 (7)	0.0787 (14)	−0.0085 (6)	0.0016 (8)	−0.0001 (8)
O17	0.0368 (9)	0.0295 (8)	0.0412 (10)	−0.0081 (7)	−0.0010 (8)	0.0006 (8)
N20	0.0227 (8)	0.0246 (8)	0.0213 (9)	−0.0011 (7)	−0.0005 (7)	0.0043 (7)
N19	0.0219 (8)	0.0250 (8)	0.0293 (10)	−0.0030 (7)	−0.0011 (7)	0.0023 (8)
C1	0.0235 (9)	0.0278 (10)	0.0236 (10)	0.0007 (8)	−0.0017 (8)	0.0047 (8)
C2	0.0270 (10)	0.0285 (10)	0.0294 (12)	−0.0016 (8)	−0.0028 (9)	0.0031 (10)
C7	0.0278 (10)	0.0228 (10)	0.0173 (9)	0.0007 (8)	−0.0001 (8)	0.0046 (8)
C4	0.0323 (11)	0.0434 (14)	0.0277 (12)	0.0092 (10)	0.0011 (10)	0.0002 (10)
C9	0.0452 (14)	0.0251 (9)	0.0239 (12)	0.0024 (10)	0.0028 (10)	0.0007 (9)
C14	0.0314 (11)	0.0358 (12)	0.0332 (14)	−0.0046 (9)	−0.0002 (10)	0.0053 (10)
C13	0.0249 (9)	0.0284 (11)	0.0276 (13)	0.0024 (8)	0.0011 (9)	0.0033 (9)
C10	0.0412 (13)	0.0333 (12)	0.0233 (12)	0.0114 (10)	0.0048 (10)	0.0022 (10)
C3	0.0360 (12)	0.0292 (11)	0.0334 (13)	0.0009 (9)	−0.0015 (11)	−0.0003 (10)
C8	0.0345 (11)	0.0271 (10)	0.0204 (11)	−0.0010 (9)	−0.0003 (9)	0.0042 (9)
C11	0.0297 (11)	0.0323 (11)	0.0200 (11)	0.0077 (9)	0.0030 (8)	0.0066 (9)
C6	0.0253 (10)	0.0307 (11)	0.0352 (13)	−0.0034 (9)	−0.0027 (10)	0.0042 (10)
C12	0.0262 (10)	0.0289 (11)	0.0161 (10)	0.0023 (8)	0.0013 (8)	0.0059 (8)
C15	0.0242 (10)	0.0528 (14)	0.0322 (14)	0.0008 (10)	−0.0003 (10)	0.0070 (12)
C16	0.0266 (11)	0.0464 (14)	0.0296 (13)	0.0115 (10)	0.0040 (10)	0.0066 (11)
C5	0.0240 (10)	0.0462 (14)	0.0358 (14)	0.0008 (10)	0.0017 (10)	0.0064 (12)

### Geometric parameters (Å, º)

**Table:**

F18—C6	1.351 (3)	C9—H27	0.95
O17—C8	1.264 (3)	C14—C13	1.383 (3)
N20—N19	1.310 (2)	C14—C15	1.393 (4)
N20—C7	1.322 (3)	C14—H26	0.95
N19—C1	1.399 (3)	C13—C12	1.395 (3)
N19—H21	0.88	C13—H28	0.95
C1—C2	1.386 (3)	C10—C11	1.430 (4)
C1—C6	1.394 (3)	C10—H29	0.95
C2—C3	1.381 (3)	C3—H23	0.95
C2—H22	0.95	C11—C12	1.411 (3)
C7—C8	1.457 (3)	C11—C16	1.413 (3)
C7—C12	1.462 (3)	C6—C5	1.373 (4)
C4—C5	1.385 (4)	C15—C16	1.368 (4)
C4—C3	1.386 (4)	C15—H30	0.95
C4—H24	0.95	C16—H31	0.95
C9—C10	1.349 (4)	C5—H25	0.95
C9—C8	1.444 (3)		
			
N19—N20—C7	118.22 (18)	C9—C10—H29	118.7
N20—N19—C1	118.27 (18)	C11—C10—H29	118.7
N20—N19—H21	120.9	C2—C3—C4	120.8 (2)
C1—N19—H21	120.9	C2—C3—H23	119.6
C2—C1—C6	118.2 (2)	C4—C3—H23	119.6
C2—C1—N19	123.5 (2)	O17—C8—C9	120.8 (2)
C6—C1—N19	118.2 (2)	O17—C8—C7	121.5 (2)
C3—C2—C1	119.9 (2)	C9—C8—C7	117.7 (2)
C3—C2—H22	120.1	C12—C11—C16	119.0 (2)
C1—C2—H22	120.1	C12—C11—C10	119.8 (2)
N20—C7—C8	124.1 (2)	C16—C11—C10	121.2 (2)
N20—C7—C12	115.91 (19)	F18—C6—C5	120.0 (2)
C8—C7—C12	119.92 (19)	F18—C6—C1	117.4 (2)
C5—C4—C3	120.2 (2)	C5—C6—C1	122.6 (2)
C5—C4—H24	119.9	C13—C12—C11	119.1 (2)
C3—C4—H24	119.9	C13—C12—C7	122.07 (19)
C10—C9—C8	121.3 (2)	C11—C12—C7	118.8 (2)
C10—C9—H27	119.3	C16—C15—C14	120.1 (2)
C8—C9—H27	119.3	C16—C15—H30	120
C13—C14—C15	120.2 (2)	C14—C15—H30	120
C13—C14—H26	119.9	C15—C16—C11	120.8 (2)
C15—C14—H26	119.9	C15—C16—H31	119.6
C14—C13—C12	120.8 (2)	C11—C16—H31	119.6
C14—C13—H28	119.6	C6—C5—C4	118.3 (2)
C12—C13—H28	119.6	C6—C5—H25	120.8
C9—C10—C11	122.5 (2)	C4—C5—H25	120.8
			
C7—N20—N19—C1	−177.5 (2)	N19—C1—C6—F18	1.0 (3)
N20—N19—C1—C2	−14.4 (3)	C2—C1—C6—C5	1.8 (4)
N20—N19—C1—C6	163.7 (2)	N19—C1—C6—C5	−176.5 (2)
C6—C1—C2—C3	−0.7 (4)	C14—C13—C12—C11	0.0 (3)
N19—C1—C2—C3	177.4 (2)	C14—C13—C12—C7	−178.1 (2)
N19—N20—C7—C8	−1.0 (3)	C16—C11—C12—C13	0.0 (3)
N19—N20—C7—C12	177.15 (19)	C10—C11—C12—C13	−179.9 (2)
C15—C14—C13—C12	0.1 (4)	C16—C11—C12—C7	178.2 (2)
C8—C9—C10—C11	0.6 (4)	C10—C11—C12—C7	−1.7 (3)
C1—C2—C3—C4	−0.8 (4)	N20—C7—C12—C13	0.5 (3)
C5—C4—C3—C2	1.3 (4)	C8—C7—C12—C13	178.7 (2)
C10—C9—C8—O17	177.6 (2)	N20—C7—C12—C11	−177.6 (2)
C10—C9—C8—C7	−1.7 (3)	C8—C7—C12—C11	0.6 (3)
N20—C7—C8—O17	−0.2 (4)	C13—C14—C15—C16	−0.3 (4)
C12—C7—C8—O17	−178.3 (2)	C14—C15—C16—C11	0.3 (4)
N20—C7—C8—C9	179.2 (2)	C12—C11—C16—C15	−0.2 (4)
C12—C7—C8—C9	1.1 (3)	C10—C11—C16—C15	179.7 (2)
C9—C10—C11—C12	1.2 (4)	F18—C6—C5—C4	−178.6 (3)
C9—C10—C11—C16	−178.7 (2)	C1—C6—C5—C4	−1.3 (4)
C2—C1—C6—F18	179.2 (2)	C3—C4—C5—C6	−0.3 (4)

### Hydrogen-bond geometry (Å, º)

**Table:**

*D*—H···*A*	*D*—H	H···*A*	*D*···*A*	*D*—H···*A*
N19—H21···O17	0.88	1.84	2.541 (3)	135
C2—H22···O17^i^	0.95	2.63	3.242 (3)	122
C5—H25···O17^ii^	0.95	2.64	3.539 (3)	159

Symmetry codes: (i) *x*, *y*−1, *z*; (ii) −*x*+1, −*y*+1, *z*−1/2.
